# Characterizing the polygenic architecture of complex traits in populations of East Asian and European descent

**DOI:** 10.1186/s40246-023-00514-3

**Published:** 2023-07-20

**Authors:** Antonella De Lillo, Frank R. Wendt, Gita A. Pathak, Renato Polimanti

**Affiliations:** 1 Department of Psychiatry, Yale University School of Medicine, 60 Temple, Suite 7A, New Haven, CT 06510 USA; 2 Department of Biology, University of Rome “Tor Vergata”, Rome, Italy; 3 Department of Anthropology, University of Toronto, Mississauga, ON Canada; 4 Biostatistics Division, Dalla Lana School of Public Health, University of Toronto, Toronto, ON Canada; 5 VA CT Healthcare Center, West Haven, CT USA; 6 Wu Tsai Institute, Yale University, New Haven, CT USA

## Abstract

To investigate the polygenicity of complex traits in populations of East Asian (EAS) and European (EUR) descents, we leveraged genome-wide data from Biobank Japan, UK Biobank, and FinnGen cohorts. Specifically, we analyzed up to 215 outcomes related to 18 health domains, assessing their polygenic architecture via descriptive statistics, such as the proportion of susceptibility SNPs per trait (*π*_*c*_). While we did not observe EAS–EUR differences in the overall distribution of polygenicity parameters across the phenotypes investigated, there were ancestry-specific patterns in the polygenicity differences between health domains. In EAS, pairwise comparisons across health domains showed enrichment for *π*_*c*_ differences related to hematological and metabolic traits (hematological fold-enrichment = 4.45, *p* = 2.15 × 10^–7^; metabolic fold-enrichment = 4.05, *p* = 4.01 × 10^–6^). For both categories, the proportion of susceptibility SNPs was lower than that observed for several other health domains (EAS-hematological median *π*_*c*_ = 0.15%, EAS-metabolic median *π*_*c*_ = 0.18%) with the strongest *π*_*c*_ difference with respect to respiratory traits (EAS-respiratory median *π*_*c*_ = 0.50%; hematological-*p* = 2.26 × 10^–3^; metabolic-*p* = 3.48 × 10^–3^). In EUR, pairwise comparisons showed multiple *π*_*c*_ differences related to the endocrine category (fold-enrichment = 5.83, *p* = 4.76 × 10^–6^), where these traits showed a low proportion of susceptibility SNPs (EUR-endocrine median *π*_*c*_ = 0.01%) with the strongest difference with respect to psychiatric phenotypes (EUR-psychiatric median *π*_*c*_ = 0.50%; *p* = 1.19 × 10^–4^). Simulating sample sizes of 1,000,000 and 5,000,000 individuals, we also showed that ancestry-specific polygenicity patterns translate into differences across health domains in the genetic variance explained by susceptibility SNPs projected to be genome-wide significant (e.g., EAS hematological-neoplasm *p* = 2.18 × 10^–4^; EUR endocrine-gastrointestinal *p* = 6.80 × 10^–4^). These findings highlight that traits related to the same health domains may present ancestry-specific variability in their polygenicity.

**Supplementary Information:**

The online version contains supplementary material available at 10.1186/s40246-023-00514-3.

## Introduction

Genome-wide association studies (GWAS) are improving our understanding of the predisposition to human traits and diseases, providing insights into their underlying biological mechanisms [1]. However, their ability to disentangle complex phenotypes is mainly proportional to the sample size of the cohorts investigated, because most human traits and diseases are characterized by a polygenic architecture (i.e., their heritability is due to the contribution of thousands of risk loci with small individual effects) [2–4]. There are differences in the degree of polygenicity among complex traits where extremely high polygenicity is observed for psychiatric disorders such as depression and relatively low polygenicity is present for physical conditions such as ulcerative colitis [5]. Multiple mechanisms are likely to contribute to this variation. Purifying selection (i.e., the selective removal of deleterious alleles across the genome) plays a major role in shaping the polygenic architecture of human traits and diseases [6, 7]. However, phenotypic heterogeneity could also contribute to the degree of polygenicity observed. Among psychiatric disorders, the number of diagnostic combinations was associated with effect size variance for trait-associated loci [8]. Understanding the dynamics shaping the polygenicity variation across the human phenotypic spectrum can generate important insights into the evolutionary basis of human traits. Additionally, defining polygenicity patterns can help to estimate more accurately the statistical power of phenotype-specific gene discovery analyses and to model more precisely polygenic scores (PGS) to stratify disease risk. Unfortunately, the majority of studies investigating polygenicity patterns across human traits and diseases are based on data generated from individuals of European descent [5–8]. Although we expect consistency in the biology of complex phenotypes among worldwide populations, there may be differences due to the evolutionary history of certain ancestry groups.

In the present study, we leveraged genome-wide association statistics previously generated [9] from Biobank Japan (BBJ) [10], UK Biobank (UKB) [11], and FinnGen [12] to estimate the effect size distribution of 215 traits comparing differences among 18 health domains in up to 178,726 and 492,803 individuals of East Asian (EAS) and European (EUR) descent, respectively (Additional file 1: Table S1). In addition to estimating the number of susceptibility single nucleotide polymorphisms (SNPs) and their effect size distribution, we also calculated the genetic variance explained by the genome-wide significant variants projected considering sample sizes of 1,000,0000 and 5,000,000 individuals. Our findings provide novel information regarding polygenicity patterns across the human phenotypic spectrum, highlighting possible similarities and differences between EAS and EUR ancestries.

## Results

### Effect-size distribution analysis

We analyzed genome-wide association statistics previously generated from BBJ, and a UKB-FinnGen meta-analysis [9]. These included up to 215 traits related to 18 health domains (Table 1; Additional file 1: Table S1). Using GENESIS (GENetic Effect-Size distribution Inference from Summary-level data) approach [5], we estimated the proportion of susceptibility SNPs per trait (*π*_*c*_), the variance parameter for non-null SNPs (*σ*^2^), and residual effects not captured by the variance of effect sizes (*a*) (Table 1; Additional file 1: Table S1). The analyses were conducted separately for EAS and EUR. To determine within- and between-population differences of *π*_*c*_, *σ*^2^, and *a* parameters, we applied the non-parametric Kruskal–Wallis (KW) test and conducted post-hoc analyses using Dunn non-parametric test for the pairwise comparisons. The application of non-parametric tests permitted us to avoid issues related to the distribution of the variables investigated and to the presence of possible outliers.

**Table 1:** Distribution (median, maximum, and minimum) of polygenicity parameters across phenotypic categories in populations of East Asian and European descent (EAS and EUR, respectively)

Category	EAS, median (max–min)				EUR, median (max–min)			
	*N*	*π*_*c*_	*σ*^2^	*a*	*N*	*π*_*c*_	*σ*^2^	*a*
Body structures	4	7.1e−3 (1e−2–4.7e−3)	2e−5 (1e−4–1e−5)	2e−6 (3e−6–1e−6)	1	5e−3	< 1e−6	2e−6
Cardiovascular	22	5e−3 (6.7e−3–6e−4)	7.4e−5 (8e−4– < 1e−6)	1e−6 (2e−5– − 2e−4)	18	5e−3 (5e−3–1.7e−3)	1e−4 (3e−4– < 1e−6)	2e−6 (1e−5– − 2e−5)
Dermatological	4	5e−3 (5.1e−3–5e−5)	< 1e−6 (1.3e−2– < 1e−6)	5e−6 (1.1e−1– − 1e−4)	4	5e−3 (5e−3–4.4e−3)	3e−4 (4e−4– < 1e−6)	− 2e−5 (− 2e−6– − 2e−4)
Ear, nose, throat	5	5e−3 (6.5e−3–5e−3)	2e−4 (6e−4– < 1e−6)	− 1e−5 (1e−5– − 1e−4)	5	5e−3 (5e−3–4e−4)	1e−4 (2.1e−3–1e−4)	1e−6 (1e−5– − 1e−5)
Endocrine	5	5e−3 (5e−3–1e−4)	4e−5 (8.9e−3– < 1e−6)	1e−5 (2e−5– − 3e−6)	5	1e−4 (1.4e−3–5e−5)	8.1e−3 (1.8e−2–4e−4)	1e−5 (2e−5–4e−6)
Environment	1	5e−3	1e−4	− 2e−6	1	5e−3	5e−5	1e−6
Gastrointestinal	8	5e−3 (5e−3–1.1e−3)	4e−5 (6e−4– < 1e−6)	1e−6 (3e−5– − 2e−5)	7	5e−3 (1.1e−2–1e−3)	3e−5 (8e−4– < 1e−6)	4e−6 (1e−5– − 3e−6)
Hematological	18	1.5e−3 (5e−3–2e−4)	1e−4 (3e−4– < 1e−6)	1e−6 (1e−5– − 1.3e−3)	4	5e−3 (5e−3–5e−3)	4e−5 (1e−4– < 1e−6)	1e−6 (3e−6– − 1e−4)
Immunological	28	5e−3 (7.1e−3–2e−5)	2e−4 (1e−2– < 1e−6)	< 1e−6 (8e−5– − 2e−4)	27	5e−3 (6.7e−3–3e−5)	1e−4 (3.6e−2– < 1e−6)	2e−6 (2e−5– − 6e−4)
Medication	22	5e−3 (6.2e−3–1e−4)	1e−4 (3.2e−3–1e−5)	1e−6 (1e−5– − 3e−5)	0	NA	NA	NA
Metabolic	31	1.8e−3 (5.2e−3–2e−5)	1e−4 (7.9e−3– < 1e−6)	1e−6 (1e−5– − 4e−5)	15	5e−3 (0.78–3e−5)	1e−4 (4.3e−2– < 1e−6)	4e−6 (1e−4– − 2e−5)
Musculoskeletal	9	5e−3 (5e−3–1e−4)	< 1e−6 (5e−3– < 1e−6)	1e−6 (5e−5– − 4e−4)	9	5e−3 (1.1e−2–1.7e−3)	5e−5 (4e−4– < 1e−6)	2e−6 (1e−5– − 4e−4)
Neoplasms	17	5e−3 (6e−3–3e−5)	3e−4 (3.2e−2– < 1e−6)	2e−6 (1e−4– − 1e−4)	17	5e−3 (5e−3–2e−5)	2e−4 (3e−2– < 1e−6)	4e−6 (3e−5– − 3e−5)
Neurological	7	5e−3 (5.2e−3–3.2e−3)	1e−4 (1.8e−3– < 1e−6)	− 1e−5 (4e−6– − 2e−4)	6	5e−3 (5e−3–3.6e−3)	1e−4 (2e−4– < 1e−6)	− 4e−6 (1e−5– − 4e−5)
Ophthalmological	8	5e−3 (5e−3–8e−4)	5e−5 (4e−4– < 1e−6)	< 1e−6 (2e−5– − 2e−5)	7	2e−3 (5e−3–3e−5)	3e−4 (2e−2– < 1e−6)	5e−6 (1e−5– − 2e−6)
Psychiatric	5	5e−3 (6.7e−3–5e−3)	2e−4 (1.8e−3– < 1e−6)	− 2e−5 (1e−5– − 2e−4)	5	5e−3 (9e−3–5e−3)	1e−4 (3e−4–1e−4)	2e−6 (3e−6– − 1e−5)
Respiratory	9	5e−3 (5e−3–5e−3)	< 1e−6 (2e−4– < 1e−6)	− 1e−5 (1e−5– − 1e−4)	9	5e−3 (8.4e−3–2e−4)	1e−4 (2.1e−3–2e−5)	− 3e−6 (1e−5– − 1e−5)
Urogenital	12	5e−3 (5.5e−3–3e−4)	4e−6 (1.5e−3– < 1e−6)	− 2e−6 (3e−5– − 1e−4)	12	5e−3 (5e−3–1e−4)	1e−4 (1.4e−3– < 1e−6)	3e−6 (4e−6– − 2e−6)

Estimates are reported using a scientific notation. Maximum and minimum estimates are not included for categories including a single trait

*π*_*c*_ The proportion of susceptibility SNPs per trait, *σ*^2^ The variance parameter for non-null SNPs, *a* Residual effects not captured by the variance of effect sizes, *NA* not available

For the majority of the domains investigated, the median *π*_*c*_ estimate was 0.50% in both EAS and EUR (Table 1). Conversely, more variability was observed with respect to the median estimates of *σ*^2^ and *a* parameters.

**Fig. 1:**
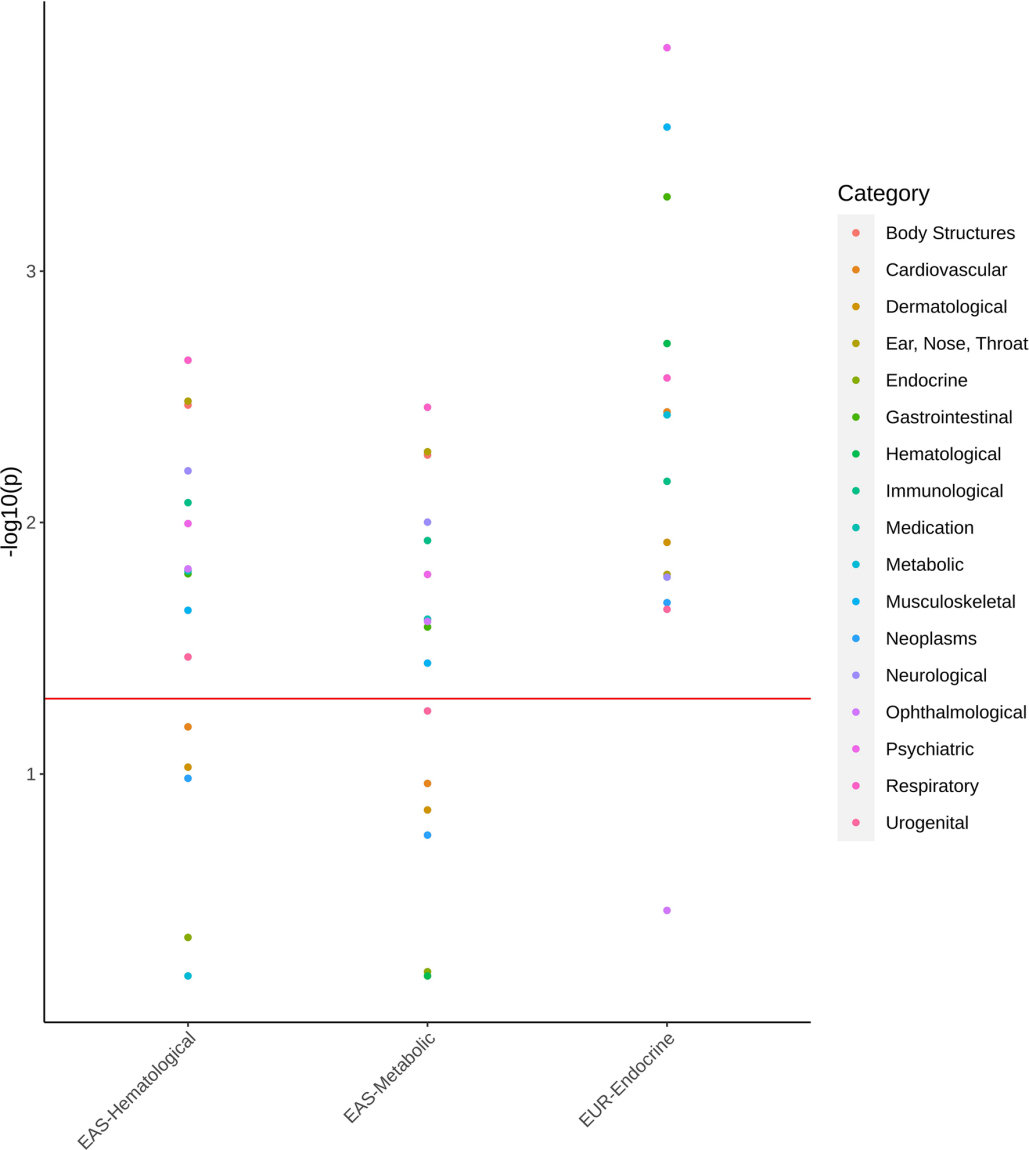
Pairwise comparison (Dunn test) of the proportion of susceptibility SNPs (*π*_*c*_) in hematological and metabolic traits assessed in populations of East Asian descent (EAS-hematological and EAS-metabolic, respectively) and in endocrine traits assessed in population of European descent (EUR-Endocrine) with respect to other phenotypic categories. Details of each comparison are available in Additional file 1: Tables S3 and S4

For the within-population analyses, we investigated whether there are differences among traits related to different phenotypic categories (Table 1; Additional file 1: Table S1). This analysis was limited to categories including at least four traits. In EAS, the proportion of susceptibility SNPs was statistically different among phenotypic categories (*π*_*c*_; KW statistic = 39.14, *p* = 6.41 × 10^–3^), while no difference was observed with respect to *σ*^2^ and *a* parameters (Additional file 1: Table S2). Although they did not survive false discovery rate correction (FDR) for multiple testing (Additional file 1: Table S3), the nominally significant pairwise comparisons showed enrichment for *π*_*c*_ differences related to hematological and metabolic traits (hematological fold-enrichment = 4.45, *p* = 2.15 × 10^–7^; metabolic fold-enrichment = 4.05, *p* = 4.01 × 10^–6^). For both categories, the proportion of susceptibility SNPs was lower than that observed for several other health domains (EAS-hematological median *π*_*c*_ = 0.15%, EAS-metabolic median *π*_*c*_ = 0.18%; Fig. 1) with the strongest *π*_*c*_ difference with respect to respiratory traits (EAS-respiratory median *π*_*c*_ = 0.50%; hematological Dunn statistic = 3.05, *p* = 2.26 × 10^–3^; metabolic Dunn statistic = 2.92, *p* = 3.48 × 10^–3^).

In EUR, significant differences among phenotypic categories were observed with respect to *π*_*c*_ (KW statistic = 35.27, *p* = 0.013) and *a* parameters (KW statistic = 31.17, *p* = 0.039), but not for *σ*^2^ (KW statistic = 25.05, *p* = 0.477). For *π*_*c*_, nine pairwise comparisons showed differences surviving multiple testing correction (FDR *q* < 0.05; Additional file 1: Table S4). Seven of them were related to the endocrine category (fold-enrichment = 5.83, *p* = 4.76 × 10^–6^), where these traits showed a low proportion of susceptibility SNPs (EUR-endocrine median *π*_*c*_ = 0.01%; Fig. 1) with the strongest difference with respect to psychiatric phenotypes (EUR-psychiatric median *π*_*c*_ = 0.50%; Dunn statistic = 3.83, *p* = 1.19 × 10^–4^). For parameter *a*, we observed four pairwise comparisons surviving FDR correction (FDR *q* < 0.05; Additional file 1: Table S5). Three of them were related to the endocrine category (EUR-endocrine median *a* = 0.001%) that showed higher residual effects than dermatological traits (EUR-dermatological median *a* =  − 0.002%; Dunn statistic =  − 3.91, *p* = 9.32 × 10^–5^), respiratory phenotypes (EUR-respiratory median *a* =  − 0.0003%; Dunn statistic =  − 3.38, *p* = 7.17 × 10^–4^), and neurological outcomes (EUR-neurological median *a* =  − 0.0004%; Dunn statistic =  − 3.30, *p* = 9.54 × 10^–4^). The dermatological category also showed lower residual effects than metabolic traits (Dunn statistic = 3.20, *p* = 1.39 × 10^–3^).

Comparing EAS and EUR, we observed a statistically significant difference with respect to residual effects not captured by the variance of effect sizes (parameter *a*: KW statistic = 8.79, *p* = 3.03 × 10^–3^), but not for *π*_*c*_ and *σ*^2^ (Additional file 1: Table S6). A category-specific EAS-EUR comparison of parameter *a* (Additional file 1: Table S7) highlighted nominally significant differences for metabolic traits (EAS median *a* = 0.0001%; EUR median *a* = 0.0004%; KW statistic = 5.22, *p* = 0.022) and urogenital phenotypes (EAS median *a* =  − 0.0002; EUR median *a* = 0.0003%; KW statistic = 4.08, *p* = 0.043). To follow up the results of the within-population analyses, we tested EAS-EUR *π*_*c*_ differences within the three categories that showed statistically significant enrichments (i.e., hematological, metabolic, and endocrine categories; Fig. 1). In EAS, hematologic traits showed a lower proportion of susceptibility SNPs than that observed in EUR (EAS median *π*_*c*_ = 0.15%, EUR median *π*_*c*_ = 0.50%, KW statistic = 5.96, *p* = 0.015). A similar trend was also observed for metabolic traits (EAS median *π*_*c*_ = 0.18%, EUR median *π*_*c*_ = 0.50%, KW statistic = 3.33, *p* = 0.068). Conversely, the endocrine category had a higher proportion of susceptibility SNPs in EAS (endocrine median *π*_*c*_ = 0.50%) compared to EUR (endocrine median *π*_*c*_ = 0.01%; KW statistic = 4.03, *p* = 0.045).

#### Projected genetic variance explained by susceptibility SNPs

To characterize further the implications of EAS and EUR polygenicity variation across phenotypic categories, we estimated the proportion of genetic variance explained by susceptibility SNPs reaching genome-wide significance considering projected sample sizes of 1,000,000 and 5,000,000 individuals (GV_%1M_ and GV_%5M_, respectively; Additional file 1: Table S8). For some traits, GV_%_ could not be calculated because of the low SNP-based heritability z-scores.

**Fig. 2:**
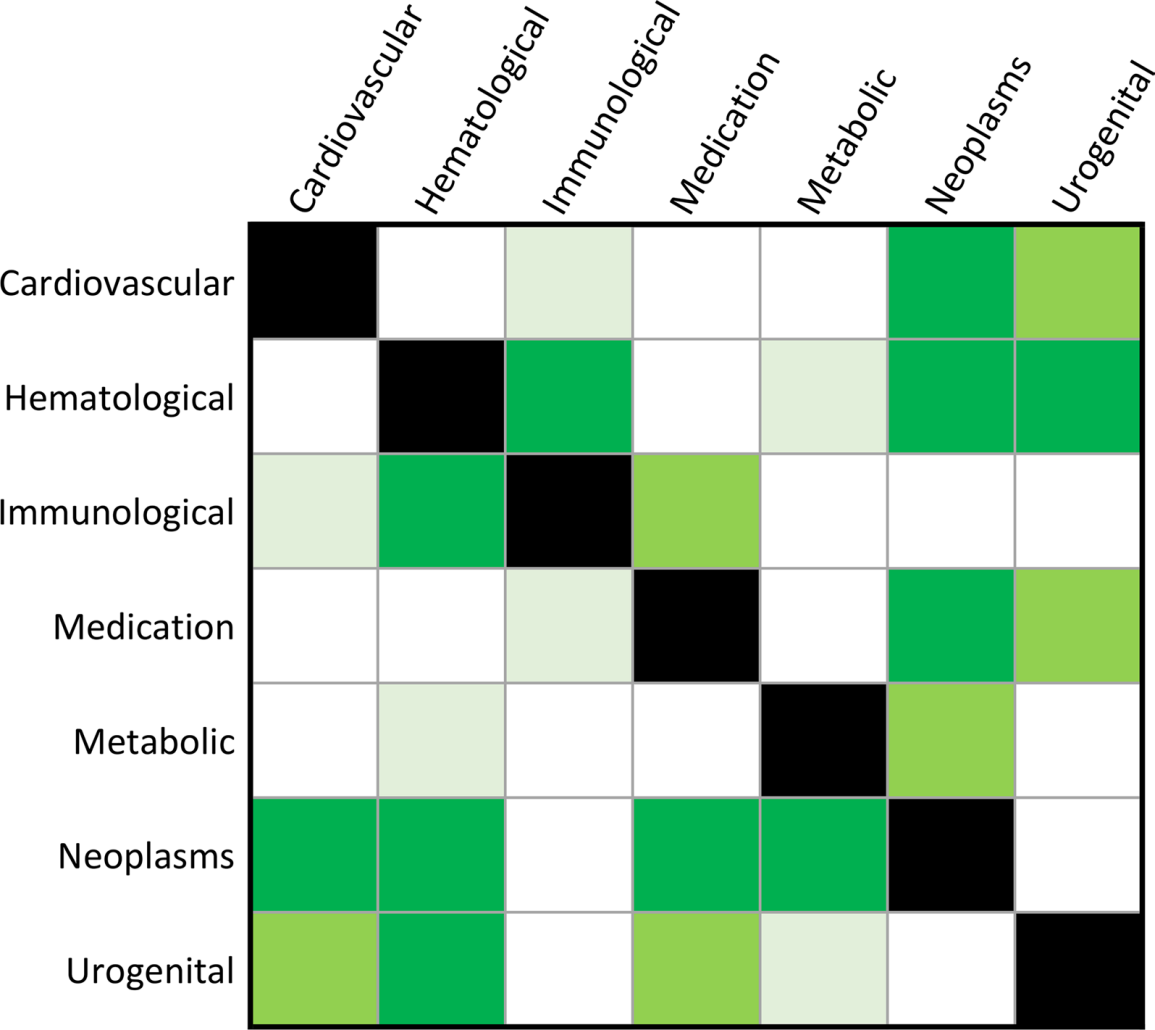
Pairwise comparison (Dunn test) among phenotypic categories of the proportion of genetic variance explained by susceptibility SNPs reaching genome-wide significance considering projected sample sizes of 1,000,000 (bottom triangle) and 5,000,000 individuals (upper triangle) of East Asian descent. Cell color corresponds to the statistical significance of the pairwise comparison: bright green (false discovery rate *q* < 0.05), green (*p* < 0.05), light green (*p* < 0.1), and white (*p* > 0.1). Details of each comparison are available in Additional file 1: Table S9

In EAS, both GV_%1M_ and GV_%5M_ showed significant differences across phenotypic categories (GV_%1M_ KW statistic = 21.95, *p* = 1.24 × 10^–3^; GV_%5M_ KW statistic = 20.91, *p* = 1.91 × 10^–3^). Results of the pairwise comparisons were consistent between the two analyses (i.e., *N* = 1,000,000 and *N* = 5,000,000) with five FDR-significant differences shared between them (FDR *q* < 0.05; Fig. 2, Additional file 1: Table S9) with the strongest one being between hematological and neoplasm categories (GV_%1M_ Dunn statistic = 3.70, *p* = 2.18 × 10^–4^; GV_%5M_ Dunn statistic = 3.50, *p* = 4.70 × 10^–4^).

In EUR, while KW test showed differences across phenotypic categories with respect to two analyses (GV_%1M_ KW statistic = 21.95, *p* = 1.24 × 10^–3^; GV_%5M_ KW statistic = 20.91, *p* = 1.91 × 10^–3^), the only FDR-significant pairwise difference was between endocrine and gastrointestinal categories (GV_%1M_ and GV_%5M_: Dunn statistic =  − 3.39, *p* = 6.80 × 10^–4^; Additional file 1: Table S10).

In the between-population comparison, we did not find a significant EAS-EUR difference with respect to GV_%1M_ and GV_%5M_ estimates considering the overall distribution of the traits available and the major phenotypic categories (*p* > 0.2; Additional file 1: Tables S11 and S12, respectively).

## Discussion

The present study leveraged BBJ, UKB, and FinnGen cohorts to investigate the polygenicity of complex traits in EAS and EUR individuals. To our knowledge, this is the first effort to comprehensively investigate polygenicity patterns across the human phenotypic spectrum in multiple ancestry groups. In line with the expectation that the biology of complex phenotypes should be consistent among worldwide populations, we found that there was no statistical difference in the proportion of susceptibility SNPs when testing the overall distribution across multiple traits. However, when investigating polygenicity variation (i.e., the proportion of susceptibility SNPs per trait) with respect to specific health domains, we observed ancestry-specific patterns.

In EAS, there was strong enrichment for metabolic and hematologic categories when testing differences with respect to the proportion of susceptibility SNPs. Specifically, metabolic and hematologic traits showed a lower degree of polygenicity (i.e., a lower proportion of susceptibility SNPs) than phenotypes related to several other categories (e.g., respiratory, neurological, psychiatric, and immunological). The polygenicity of complex traits is primarily driven by purifying selection where new mutations with large effects tend to be removed from the population while variants with small effects are more likely to become common [6, 7]. Ancestry-specific evolutionary dynamics may be responsible for the low polygenicity of metabolic and hematologic traits. Compared to other ancestry groups, metabolic risk in EAS populations appears to be stronger where individuals tend to develop prediabetes and diabetes at a younger age and at a lower body mass index and waist circumference [13, 14]. The genetic risk of type-2 diabetes is partially shared between EAS and EUR, but there may be EAS-specific pathways related to skeletal muscle, adipose, and liver development and function [15]. This ancestry-specific genetic risk may be due to adaptations to cereal-based diets. Indeed, the diet of EAS populations relied on wild and domesticated rice for more than 10,000 years [16, 17]. This long exposure to a cereal associated with high glucose load may be responsible for the signatures of selective sweeps and polygenic adaption observed in Chinese, Korean, and Japanese populations in genes involved in fatty acids metabolism, cholesterol/triglycerides biosynthesis from carbohydrates, regulation of glucose homeostasis, and production of retinoic acid [18].

With respect to the lower polygenicity of hematologic traits in EAS compared to other health domains, there is limited information regarding which dynamics may be responsible. However, there is consistent literature regarding the impact of human evolutionary history on shaping the variation of genes related to hematologic phenotypes. For example, adaptation to malaria strongly influenced the genetics of hematologic traits through a systematic positive selection of protective alleles that is likely to be partially different across ancestry groups [19]. Denisovan-introgressed alleles were responsible for high-altitude adaption in Tibetans, which showed a modified response to hypoxia-altering changes in hemoglobin concentration [20]. Denisovan-introgressed alleles were also associated with hematologic traits (e.g., albumin-globulin ratio) in EAS [21]. Blood biomarkers showed strong cross-ancestry heterogeneity in the effect of genome-wide significant loci [22]. While these mechanisms do not directly explain the lower polygenicity of hematologic traits in EAS, they support that blood-related phenotypes played an important role in human evolution and that there may be specific adaptation processes in EAS populations affecting them.

In EUR, endocrine traits showed a lower polygenicity degree than other complex phenotypes (i.e., psychiatric, musculoskeletal, gastrointestinal, hematological, respiratory, cardiovascular, and metabolic traits). The endocrine system plays a key role in many aspects (e.g., development, reproduction, and response to the environment) that were essential to the success of the human species [23]. Indeed, several studies reported evidence of signatures of multiple selective pressures acting in genes related to the endocrine system [24–26]. In EUR, recent positive directional selection was observed in human male reproductive genes in response to different environmental conditions [27]. A Neanderthal introgressed allele increasing the levels of progesterone receptor is associated with having more siblings, fewer miscarriages, and less bleeding during early pregnancy in EUR individuals [28]. Dietary changes may have also contributed to shaping hormonal responses such as the effect of agriculture introduction on thyroid hormones [29]. The presence of multiple adaption processes may have reduced the effect of purifying selection acting on endocrine-related genes in EUR.

In our study, we used genome-wide association statistics generated from the meta-analysis of UKB and FinnGen data to maximize the sample size and obtain information regarding as many phenotypes as those available in BBJ. UKB-FinnGen GWAS meta-analyses have been performed by several studies [30–33], because Finnish population is within EUR genetic variation [34] although they have a peculiar demographic history that can permit the study of alleles that are rare in other EUR populations [12].

Beyond the link between human evolution and the genetics of complex traits, the ancestry-specific polygenicity patterns have implications for the translation of genetic information into clinical care. Indeed, there are ongoing efforts into defining PGS to stratify disease risk [35]. For certain health outcomes, PGS power to stratify individual risk appears to be comparable to some monogenic mutations [36]. One of the main limitations in PGS application is modeling polygenic risk across ancestry groups due to the limited representativeness of worldwide populations among large-scale GWAS [37]. While methods are being developed to perform effectively cross-population polygenic prediction across ancestry groups [38, 39], our findings highlight that modeling polygenicity may need to account for ancestry-specific differences across different health domains. Specifically, the genetic variance explained by susceptibility SNPs projected with respect to the sample sizes of 1,000,000 and 5,000,000 individuals shows how future GWAS may generate extremely powerful PGS with ancestry-specific predictivity variation with respect to certain phenotypic categories. Our results point to several health domains that may require closer attention to cross-ancestry effects.

Most of our findings were related to the proportion of susceptibility SNPs per trait, which is informative of the polygenicity degree of the phenotypes investigated. However, we also identified significant between- and within-population differences in the residual effects not captured by the variance of effect sizes (i.e., parameter *a*). This reflects the potential systematic bias in variance estimates due to effects such as population stratification or cryptic relatedness [5]. In line with the fact that current methods can adequately control these GWAS confounders, we observed extremely low estimates of parameter *a* (Table 1). Nevertheless, we saw differences between ancestries and between categories. This is likely due to the characteristics of the cohorts and/or the populations investigated. The between-category differences may be related to the specific dynamics linking population structure to the assessment of traits analyzed. As mentioned, the systematic bias detected is extremely small. However, future larger GWAS with the statistical power to detect very small effects may need to control further the residual bias of certain confounders. While *π*_*c*_ and *a* estimates showed differences between ancestries and between categories, we did not observe significant results with respect to *σ*^2^ parameter (i.e., the variance parameter for non-null SNPs). This suggests that ancestry and category differences are primarily driven by variability in the proportion of susceptibility SNPs per trait. Further studies will be needed to confirm this hypothesis across more diverse cohorts and a large number of phenotypes.

In conclusion, we provide the first comprehensive evidence of polygenicity patterns among human traits and diseases in EAS and EUR. Our findings confirm that there is an overall similarity between ancestry groups in the polygenicity of complex phenotypes. However, we observed ancestry-specific patterns in the polygenicity observed across multiple health domains. We hypothesize that these are due to evolutionary mechanisms that acted specifically in certain population groups. These may have important implications in the future applications of PGS to individuals of diverse ancestral backgrounds. Although these are novel insights into the genetics of complex traits, we have to acknowledge several limitations. First, our study compared two ancestry groups leveraging data from three large biobanks. Because of the differences in the characteristics and the assessment of the cohorts investigated, our results may be partially due to cohort-specific factors rather than due to genetic variation between ancestries. Our study was based on publicly available datasets and we were not able to find additional resources to expand our analyses to other cohorts. Our findings will need to be confirmed in other large cohorts including diverse participants. Second, the difference in the sample size available for EAS and EUR may have affected the results of some of the analyses conducted. We hope that in the next years, additional data will become available for EAS and other population groups that are currently underrepresented in genetic research. Third, although we greatly expanded the number of phenotypes investigated with respect to the ones analyzed in the initial GENESIS analysis [5] and provided novel insights into ancestry differences, future studies will need to focus on possible differences between effect size distribution derived from single cohorts and those detected from GWAS meta-analysis of many cohorts with different characteristics. Fourth, we discussed our results in the context of EAS and EUR evolutionary history. However, because of the lack of information regarding the implication of evolutionary processes with respect to human traits and diseases across worldwide populations, some of our hypotheses should be considered speculative because they rely on findings related to a single phenotype rather than multiple phenotypes within different health domains.

## Materials and methods

### Study populations

In our study, we used data generated from EAS and EUR participants enrolled in BBJ, UKB, and FinnGen. BBJ is a prospective biobank that collected DNA and serum samples from 12 medical institutions in Japan and recruited approximately 200,000 participants, mainly of Japanese ancestry. The mean age of participants at recruitment was 63 years old, and 46% were female. BBJ phenotype information including disease endpoints, past medical history, electronic health records (EHR), biomarkers, and prescription category [10]. UKB is a biobank that enrolled more than 500,000 participants assessed through detailed web-based questionnaires on their diet, cognitive function, work history, health status, and other relevant phenotypes [11]. EHRs are available for the UKB cohort, providing information regarding primary care, hospital episodes, death registry, and laboratory test results [11]. The mean age of UKB participants at recruitment was 57 years old, and 54% were female. FinnGen is a public–private partnership project combining genotype data from Finnish biobanks and digital health record data from Finnish health registries [12]. The mean age of participants at the time of the DNA sample collection was 52 years old and 56% were female.

To avoid biases due to population stratification [40], BBJ genome-wide analyses were restricted to 178,726 EAS participants as estimated by the principal component analysis-based sample selection criteria [9]. The same approach was used to define EUR participants from UKB [9]. Principal component analysis was also used to detect ancestry outliers among FinnGen participants (description available at https://finngen.gitbook.io/documentation/v/r3/methods/phewas/quality-checks). A total of 357,658 and 135,638 EUR participants were investigated from UKB and FinnGen.

The genome-wide association statistics were generated following the analytic approach described previously [9]. Briefly, the association analysis of binary traits (i.e., disease endpoints and medication usage) was performed by using the generalized linear mixed model implemented SAIGE (v.0.37) [41], including age, age^2^, sex, age × sex, age^2^ × sex and the top 20 principal components as covariates. For sex-specific diseases, age, age^2^, and the top 20 within ancestry principal components were included as covariates, and only controls of the sex to which the disease is specific were used. BOLT-LMM (v.2.3.4) [42] was used to conduct GWAS of quantitative traits (i.e., biomarkers) by using a linear mixed model and including the same covariates as used in the binary traits above. UKB and FinnGen GWAS meta-analyzed using the inverse-variance method to create a single EUR dataset [9]. A total of 215 traits and 152 matching phenotypes were investigated in EAS and EUR, respectively (Additional file 1: Table S1).

The genome-wide association statistics used in the present study were downloaded from https://pheweb.jp/downloads in November 2021.

### Effect size distribution

The GENESIS R package [5] was used to determine the descriptive statistics regarding the effect size distribution of complex traits in EAS and EUR (Additional file 1: Table S1). Briefly, GENESIS approach can distinguish susceptibility SNPs (i.e., those that have a detectable influence without requiring genome-wide significance) from null SNPs (i.e., those that have no detectable effect on a trait) to estimate parameters describing the polygenic architecture of a trait: (i) *π*_*c*,_ the proportion of susceptibility SNPs per trait; (ii) *σ*^2^, the variance of non-null SNPs; and (iii) a, the residual effects not captured by the variance of effect-sizes (e.g., population stratification, underestimated effects of extremely small effect size SNPs, and/or genomic deflation) [5]. As recommended by the developers [5], the GWAS data used in the present study were filtered to include only HapMap3 SNPs with an ancestry-specific minor allele frequency ≥ 0.05 [43]. Using the *preprocessing()* function, SNPs were also removed if: (i) their effective sample sizes were less than 0.67 times the 90th percentile of the per-SNP sample size distribution; (ii) they were within the major histocompatibility region (excluded because of its complex LD structure); and (iii) they had extremely large effect sizes (per-SNP effect *χ*^2^ > 80). In line with GENESIS developers’ recommendations [5], we applied these filters to avoid that the effect-size distribution estimates may be affected by the presence of outlier variants. After these quality control steps, the *genesis()* function was used to implement the two-component model, which assumes that the distribution of effects for non-null SNPs follows a single normal distribution. The same preprocessing (Additional file 2: Fig. S1) and parameter estimation were performed for EAS and EUR datasets. EAS and EUR LD score reference panels were used with respect to the corresponding ancestry group (available at https://github.com/yandorazhang/GENESISasian and https://github.com/yandorazhang/GENESIS, respectively). According to simulation analyses [5], models with more than two components could substantially underestimate the total number of non-null SNPs for GWAS of modest sample size (*N* < 25,000) and this bias can some bias can persist even in much larger sample sizes (e.g., *N* = 100,000). For this reason, we decided to implement only the two-component model to avoid possible biases due to the sample size of the input GWAS.

The expected proportion of genetic variance explained by susceptibility SNPs reaching genome-wide significance considering projected sample sizes equal to 1,000,000 and 5,000,000 by applying the *projection()* function in GENESIS [5]. These sample sizes were defined, because several GWAS already included more than 1,000,000 individuals [44–50] and there is a continuing increase in GWAS sample size [1].

### Statistical analyses

We tested within- and between-population differences of the descriptive statistics related to the polygenic architecture of complex traits using non-parametric tests. This permitted us to avoid issues related to the distribution of the variables investigated and to the presence of possible outliers. The KW test was used to compare differences across multiple groups (e.g., phenotypic categories) in a single analysis. To follow up KW results, we used the Dunn test to perform post-hoc pairwise comparisons. To account for the number of pairwise comparisons, we applied FDR multiple testing correction. KW and Dunn tests were performed using the rstatix R package.

## Supplementary Information

**Additional file 1:** Supplemental Tables.

**Additional file 2: Fig. S1**. GENESIS workflow of GWAS data preprocessing.

## Data Availability

All data generated during this study are included in this published article and its Additional files.

## Abbreviations

BBJ: Biobank Japan
EAS: East Asian
EHR: Electronic health records
EUR: European
FDR: False discovery rate
GENESIS: Genetic effect-size distribution inference from summary-level data
GV%: Proportion of genetic variance explained by susceptibility SNPs projected to reach genome-wide significance
GWAS: Genome-wide association studies
KW: Kruskal–Wallis
LD: Linkage disequilibrium
PGS: Polygenic score
UKB: UK biobank

## References

[1] Abdellaoui A, Yengo L, Verweij KJH, Visscher PM (2023). 15 years of GWAS discovery: realizing the promise. Am J Hum Genet.

[2] Loh PR, Bhatia G, Gusev A, Finucane HK, Bulik-Sullivan BK, Pollack SJ (2015). Contrasting genetic architectures of schizophrenia and other complex diseases using fast variance-components analysis. Nat Genet.

[3] Johnson R, Burch KS, Hou K, Paciuc M, Pasaniuc B, Sankararaman S (2021). Estimation of regional polygenicity from GWAS provides insights into the genetic architecture of complex traits. PLoS Comput Biol.

[4] Uricchio LH (2020). Evolutionary perspectives on polygenic selection, missing heritability, and GWAS. Hum Genet.

[5] Zhang Y, Qi G, Park JH, Chatterjee N (2018). Estimation of complex effect-size distributions using summary-level statistics from genome-wide association studies across 32 complex traits. Nat Genet.

[6] Zeng J, de Vlaming R, Wu Y, Robinson MR, Lloyd-Jones LR, Yengo L (2018). Signatures of negative selection in the genetic architecture of human complex traits. Nat Genet.

[7] O'Connor LJ, Schoech AP, Hormozdiari F, Gazal S, Patterson N, Price AL (2019). Extreme polygenicity of complex traits is explained by negative selection. Am J Hum Genet.

[8] Wendt FR, Pathak GA, Overstreet C, Tylee DS, Gelernter J, Atkinson EG (2021). Characterizing the effect of background selection on the polygenicity of brain-related traits. Genomics.

[9] Sakaue S, Kanai M, Tanigawa Y, Karjalainen J, Kurki M, Koshiba S (2021). A cross-population atlas of genetic associations for 220 human phenotypes. Nat Genet.

[10] Ishigaki K, Akiyama M, Kanai M, Takahashi A, Kawakami E, Sugishita H (2020). Large-scale genome-wide association study in a Japanese population identifies novel susceptibility loci across different diseases. Nat Genet.

[11] Bycroft C, Freeman C, Petkova D, Band G, Elliott LT, Sharp K (2018). The UK Biobank resource with deep phenotyping and genomic data. Nature.

[12] Kurki MI, Karjalainen J, Palta P, Sipila TP, Kristiansson K, Donner KM (2023). FinnGen provides genetic insights from a well-phenotyped isolated population. Nature.

[13] Vicks WS, Lo JC, Guo L, Rana JS, Zhang S, Ramalingam ND (2022). Prevalence of prediabetes and diabetes vary by ethnicity among U.S. Asian adults at healthy weight, overweight, and obesity ranges: an electronic health record study. BMC Public Health.

[14] Ramachandran A, Snehalatha C, Shetty AS, Nanditha A (2012). Trends in prevalence of diabetes in Asian countries. World J Diabetes.

[15] Spracklen CN, Horikoshi M, Kim YJ, Lin K, Bragg F, Moon S (2020). Identification of type 2 diabetes loci in 433,540 East Asian individuals. Nature.

[16] Silva F, Weisskopf A, Castillo C, Murphy C, Kingwell-Banham E, Qin L (2018). A tale of two rice varieties: modelling the prehistoric dispersals of japonica and proto-indica rices. The Holocene.

[17] Jiang L, Liu L (2015). New evidence for the origins of sedentism and rice domestication in the Lower Yangzi River. China Antiquity.

[18] Landini A, Yu S, Gnecchi-Ruscone GA, Abondio P, Ojeda-Granados C, Sarno S (2021). Genomic adaptations to cereal-based diets contribute to mitigate metabolic risk in some human populations of East Asian ancestry. Evol Appl.

[19] Network MGE (2019). Insights into malaria susceptibility using genome-wide data on 17,000 individuals from Africa, Asia and Oceania. Nat Commun.

[20] Zhang X, Witt KE, Banuelos MM, Ko A, Yuan K, Xu S (2021). The history and evolution of the Denisovan-EPAS1 haplotype in Tibetans. Proc Natl Acad Sci U S A.

[21] Koller D, Wendt FR, Pathak GA, De Lillo A, De Angelis F, Cabrera-Mendoza B (2022). Denisovan and Neanderthal archaic introgression differentially impacted the genetics of complex traits in modern populations. BMC Biol.

[22] De Lillo A, D'Antona S, Pathak GA, Wendt FR, De Angelis F, Fuciarelli M (2021). Cross-ancestry genome-wide association studies identified heterogeneous loci associated with differences of allele frequency and regulome tagging between participants of European descent and other ancestry groups from the UK Biobank. Hum Mol Genet.

[23] Trumble BC, Jaeggi AV, Gurven M (2015). Evolving the neuroendocrine physiology of human and primate cooperation and collective action. Philos Trans R Soc Lond B Biol Sci.

[24] Saitou M, Resendez S, Pradhan AJ, Wu F, Lie NC, Hall NJ (2021). Sex-specific phenotypic effects and evolutionary history of an ancient polymorphic deletion of the human growth hormone receptor. Sci Adv.

[25] Bergey CM, Lopez M, Harrison GF, Patin E, Cohen JA, Quintana-Murci L (2018). Polygenic adaptation and convergent evolution on growth and cardiac genetic pathways in African and Asian rainforest hunter-gatherers. Proc Natl Acad Sci U S A.

[26] Li J, Hong X, Mesiano S, Muglia LJ, Wang X, Snyder M (2018). Natural selection has differentiated the progesterone receptor among human populations. Am J Hum Genet.

[27] Schaschl H, Wallner B (2020). Population-specific, recent positive directional selection suggests adaptation of human male reproductive genes to different environmental conditions. BMC Evol Biol.

[28] Zeberg H, Kelso J, Paabo S (2020). The neandertal progesterone receptor. Mol Biol Evol.

[29] Chekalin E, Rubanovich A, Tatarinova TV, Kasianov A, Bender N, Chekalina M (2019). Changes in biological pathways during 6000 years of civilization in Europe. Mol Biol Evol.

[30] Campos AI, Kho P, Vazquez-Prada KX, Garcia-Marin LM, Martin NG, Cuellar-Partida G (2021). Genetic susceptibility to pneumonia: A GWAS meta-analysis between the UK Biobank and FinnGen. Twin Res Hum Genet.

[31] Fairfield CJ, Drake TM, Pius R, Bretherick AD, Campbell A, Clark DW (2022). Genome-wide analysis identifies gallstone-susceptibility loci including genes regulating gastrointestinal motility. Hepatology.

[32] Gharahkhani P, Jorgenson E, Hysi P, Khawaja AP, Pendergrass S, Han X (2021). Genome-wide meta-analysis identifies 127 open-angle glaucoma loci with consistent effect across ancestries. Nat Commun.

[33] Levey DF, Stein MB, Wendt FR, Pathak GA, Zhou H, Aslan M (2021). Bi-ancestral depression GWAS in the Million Veteran Program and meta-analysis in >1.2 million individuals highlight new therapeutic directions. Nat Neurosci.

[34] Novembre J, Johnson T, Bryc K, Kutalik Z, Boyko AR, Auton A (2008). Genes mirror geography within Europe. Nature.

[35] Lewis CM, Vassos E (2020). Polygenic risk scores: from research tools to clinical instruments. Genome Med.

[36] Khera AV, Chaffin M, Aragam KG, Haas ME, Roselli C, Choi SH (2018). Genome-wide polygenic scores for common diseases identify individuals with risk equivalent to monogenic mutations. Nat Genet.

[37] Duncan L, Shen H, Gelaye B, Meijsen J, Ressler K, Feldman M (2019). Analysis of polygenic risk score usage and performance in diverse human populations. Nat Commun.

[38] Ruan Y, Lin YF, Feng YA, Chen CY, Lam M, Guo Z (2022). Improving polygenic prediction in ancestrally diverse populations. Nat Genet.

[39] Zhao Z, Fritsche LG, Smith JA, Mukherjee B, Lee S (2022). The construction of cross-population polygenic risk scores using transfer learning. Am J Hum Genet.

[40] Constantinescu AE, Mitchell RE, Zheng J, Bull CJ, Timpson NJ, Amulic B (2022). A framework for research into continental ancestry groups of the UK Biobank. Hum Genomics.

[41] Zhou W, Nielsen JB, Fritsche LG, Dey R, Gabrielsen ME, Wolford BN (2018). Efficiently controlling for case-control imbalance and sample relatedness in large-scale genetic association studies. Nat Genet.

[42] Loh PR, Tucker G, Bulik-Sullivan BK, Vilhjalmsson BJ, Finucane HK, Salem RM (2015). Efficient Bayesian mixed-model analysis increases association power in large cohorts. Nat Genet.

[43] Altshuler DM, Gibbs RA, Peltonen L, Altshuler DM, Gibbs RA, International HapMap C (2010). Integrating common and rare genetic variation in diverse human populations. Nature.

[44] Okbay A, Wu Y, Wang N, Jayashankar H, Bennett M, Nehzati SM (2022). Polygenic prediction of educational attainment within and between families from genome-wide association analyses in 3 million individuals. Nat Genet.

[45] Saunders GRB, Wang X, Chen F, Jang SK, Liu M, Wang C (2022). Genetic diversity fuels gene discovery for tobacco and alcohol use. Nature.

[46] Yengo L, Vedantam S, Marouli E, Sidorenko J, Bartell E, Sakaue S (2022). A saturated map of common genetic variants associated with human height. Nature.

[47] Graham SE, Clarke SL, Wu KH, Kanoni S, Zajac GJM, Ramdas S (2021). The power of genetic diversity in genome-wide association studies of lipids. Nature.

[48] Huang J, Huffman JE, Huang Y, Do Valle I, Assimes TL, Raghavan S (2022). Genomics and phenomics of body mass index reveals a complex disease network. Nat Commun.

[49] Liu H, Doke T, Guo D, Sheng X, Ma Z, Park J (2022). Epigenomic and transcriptomic analyses define core cell types, genes and targetable mechanisms for kidney disease. Nat Genet.

[50] Watanabe K, Jansen PR, Savage JE, Nandakumar P, Wang X (2022). Genome-wide meta-analysis of insomnia prioritizes genes associated with metabolic and psychiatric pathways. Nat Genet.

