# Supplementary figures and images for: Characterizing the polygenic architecture of complex traits in populations of East Asian and European descent
Source: Hum Genomics. 2023 Jul 20;17:67. doi: 10.1186/s40246-023-00514-3 (PMC10360343; doi:10.1186/s40246-023-00514-3)

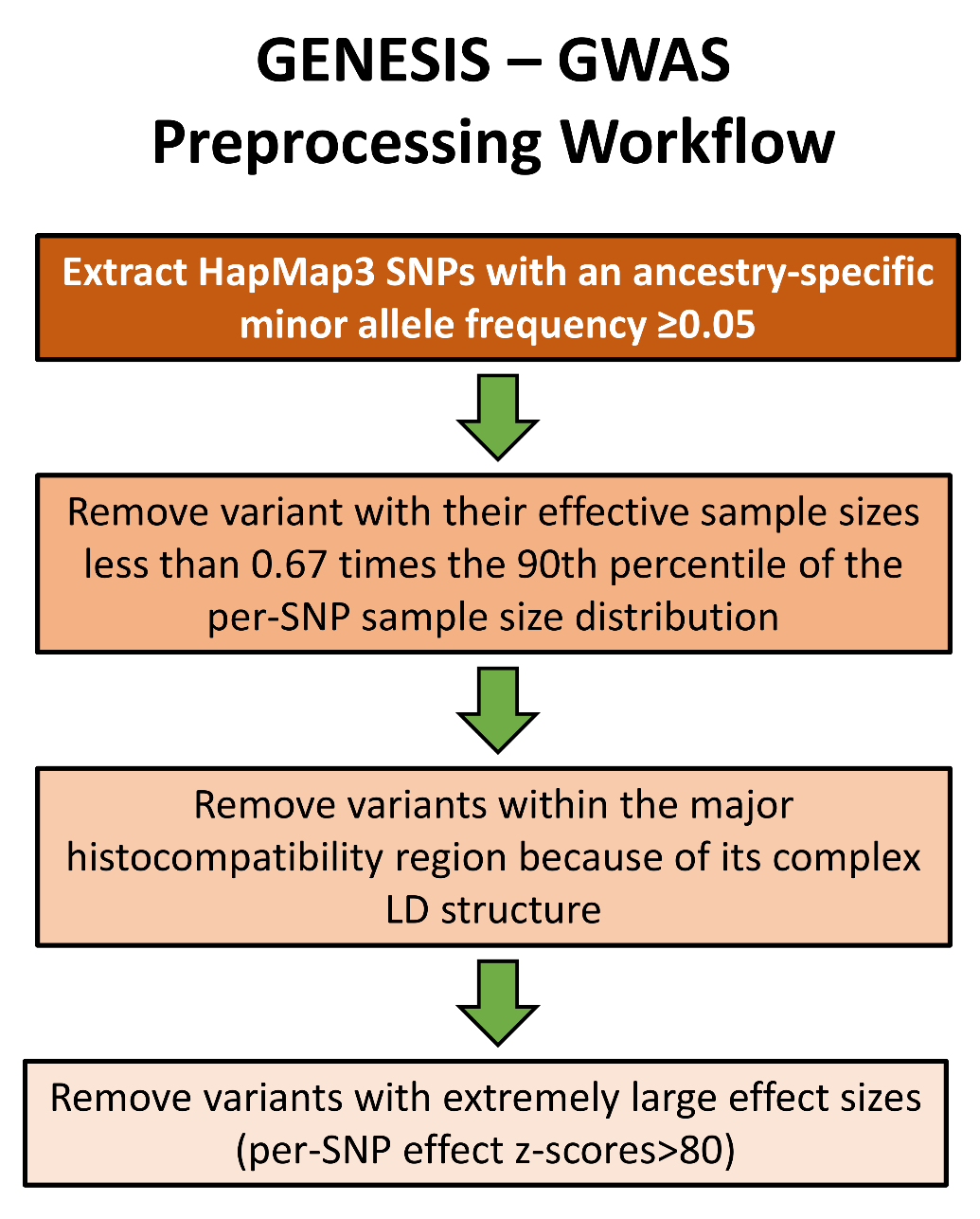


**Supplemental Figure 1**: GENESIS workflow of GWAS data preprocessing.

Supplement: Supplementary file 2 — Additional file 2: Fig. S1. GENESIS workflow of GWAS data preprocessing. [file 40246_2023_514_MOESM2_ESM.docx]
